# The role of sex and body weight on the metabolic effects of high-fat diet in C57BL/6N mice

**DOI:** 10.1038/nutd.2017.6

**Published:** 2017-04-10

**Authors:** C Ingvorsen, N A Karp, C J Lelliott

**Affiliations:** 1 Mouse Pipelines, Wellcome Trust Sanger Institute, Wellcome Trust Genome Campus, Cambridge, UK; 2 University of Cambridge Metabolic Research Laboratories, Wellcome Trust-MRC Institute of Metabolic Science, Addenbrooke's Hospital, Robinson Way, Cambridge, UK; 3 Mouse Informatics Group, Wellcome Trust Sanger Institute, Cambridge, UK

## Abstract

**Background:**

Metabolic disorders are commonly investigated using knockout and transgenic mouse models on the C57BL/6N genetic background due to its genetic susceptibility to the deleterious metabolic effects of high-fat diet (HFD). There is growing awareness of the need to consider sex in disease progression, but limited attention has been paid to sexual dimorphism in mouse models and its impact in metabolic phenotypes. We assessed the effect of HFD and the impact of sex on metabolic variables in this strain.

**Methods:**

We generated a reference data set encompassing glucose tolerance, body composition and plasma chemistry data from 586 C57BL/6N mice fed a standard chow and 733 fed a HFD collected as part of a high-throughput phenotyping pipeline. Linear mixed model regression analysis was used in a dual analysis to assess the effect of HFD as an absolute change in phenotype, but also as a relative change accounting for the potential confounding effect of body weight.

**Results:**

HFD had a significant impact on all variables tested with an average absolute effect size of 29%. For the majority of variables (78%), the treatment effect was modified by sex and this was dominated by male-specific or a male stronger effect. On average, there was a 13.2% difference in the effect size between the male and female mice for sexually dimorphic variables. HFD led to a significant body weight phenotype (24% increase), which acts as a confounding effect on the other analysed variables. For 79% of the variables, body weight was found to be a significant source of variation, but even after accounting for this confounding effect, similar HFD-induced phenotypic changes were found to when not accounting for weight.

**Conclusion:**

HFD and sex are powerful modifiers of metabolic parameters in C57BL/6N mice. We also demonstrate the value of considering body size as a covariate to obtain a richer understanding of metabolic phenotypes.

## Introduction

The development of transgenic and knockout mouse technologies has facilitated the production of *in vivo* models that have greatly enhanced our understanding of disease mechanisms and have become commonplace in medical research. The C57BL/6N (B6N) background strain is becoming the standard background for genetic manipulation of the mouse genome.^1, 2^ Similar to the well-known C57BL/6J strain, B6N mice are susceptible to high-fat diet (HFD)-induced conditions including body weight gain, increased adipose mass, liver lipid infiltration and alterations in biochemical variables.^1, 3^ Because of the increasing adoption of this background for genetically modified and non-genetically modified studies in mice, baseline data from C57BL/6N mice are important for being able to examine its performance between laboratories and to justify the use of this strain when designing experimental procedures.

Historically, research into biological processes has overlooked the impact of sex. Systematic reviews of animal research studies identified a vast over-representation of male mice, and where both sexes were studied, two-thirds of the time, the results were not statistically analysed with regard to the effect of sex.^4, 5, 6^ The observations that many human diseases exhibit some sex differences in prevalence,^7^ course, severity^8^ and drug reaction^9^ has led to a growing concern over the sex imbalance and lack of generalisability of results in biomedical research.^10^ Sexual dimorphism can be defined as the difference in a particular characteristic between sexes in either standard conditions, or in response to a stimulus. Assessment of sexual dimorphism under standard or non-challenged conditions can be thought of as binary (for example, the change in sex leads to the feature being present or absent; or if continuous trait larger or smaller). In contrast, assessing sexually dimorphic responses to stimuli is more complex with multiple potential outcomes: both sexes show similar responses; only one sex is affected by the stimulus; both sexes are affected to differing degrees; and sexes have opposite responses to a stimulus. Despite being a well-known phenomenon, to date, we have only identified a few publications that explicitly looked at the role of sex in the response to HFD in mice.^11, 12, 13, 14^

Typically with metabolic studies, the treatment will be associated with a weight change. However, body weight is known to be a powerful co-variable and this correlation can lead to an association that is true but potentially biologically misleading. The importance of considering weight in interpreting phenotypes, and doing so correctly via regression, has been raised previously.^15, 16, 17^ Therefore, a dual analysis approach has been proposed, first assessing the treatment impact on the absolute phenotype and then assessing the treatment impact on the phenotype relative to body weight.^18^ This dual analysis strategy will give a more detailed understanding of the impact of the treatment.

The International Mouse Phenotyping Consortium (IMPC^19^) aims to phenotype knockouts for all protein coding genes in the mouse genome, and provide the lines and data to form an open resource to the community. These high-throughput phenotyping pipelines are designed to systematically test mice through a number of assays in a standardised, unbiased manner to explore a range of vertebrate biology. To provide a baseline for statistical analysis of mutant phenotypes, the pipelines typically phenotype control mice matched for age, sex and strain every week. Cumulatively, this wild-type data set provides a substantial amount of information on systematically phenotyped mice in numbers that are unusual in hypothesis-driven mouse phenotyping. These data can provide insights into physiology otherwise undetected under normal laboratory conditions. The Wellcome Trust Sanger Institute Mouse Genetics Project (MGP) has been running a high-throughput phenotyping pipeline since 2006.^20^ The programme consists of standardised and validated screens, but due to changing objectives, the diet changed from a HFD to a breeders chow, giving large quantities of B6N control data under the two conditions.

In this paper, we explore the B6N data sets of the MGP to answer three questions. First, we capture the typical metabolic profile for B6N mice under standard chow diet and HFD conditions, using linear mixed model regression to fit an absolute change model (ACM) to derive the absolute changes generated by the HFD. Then, we fitted a weight-adjusted model (WAM) to assess the influence of HFD on metabolic variables after adjusting for a potential confounding effect of body weight. Then in both models, we asked whether there are sexually dimorphic differences in the metabolic response to a HFD. As a resource to the community, we provide the data and scripts.^21^

## Methods

### Animals, housing and husbandry

B6N mice were phenotyped in weekly batches of seven males and females as part of the standardised pipelines from the MGP at Wellcome Trust Sanger Institute. Mice phenotyped by the Mouse GP pipeline (January 2011–February 2012) were given a HFD from 4 weeks of age (Western RD, 829100, 21.4% crude fat content, 42% kcal as fat, ~0.2% cholesterol, Special Diet Services, Witham, UK).^20^ Mice phenotyped by the MGP Select pipeline (February 2012–February 2013) were given a breeders chow (Mouse Breeder Diet 5021, 9% crude fat content, 21% kcal as fat, 0.276 p.p.m. cholesterol, Labdiet, London, UK) from weaning. Diet compositions are given in Table 1. All mice were given water and diet *ad libitum*, unless otherwise stated.

The care and use of mice in the Wellcome Trust Sanger Institute study was carried out in accordance with UK Home Office regulations, UK Animals (Scientific Procedures) Act of 1986 under two UK Home Office licences that approved this work, which were reviewed regularly by the Wellcome Trust Sanger Institute Animal Welfare and Ethical Review Body.

### Phenotyping screen

Data from the following screens were included for analysis in this manuscript: overnight fast followed by intraperitoneal glucose tolerance test (ipGTT) at 13 weeks of age; dual-energy X-ray absorptiometry (DEXA) at 14 weeks of age; and clinical chemistry at 16 weeks of age. The two phenotyping pipelines were identical with the exception that the Mouse GP has four additional screens (hair phenotyping, open field, hot plate and stress-induced hypothermia tests). The analysis presented here assumes that the omission of these screens in the MGP Select pipeline does not impact the outcomes of the ipGTT, DEXA and clinical chemistry screens. The final data set contained 296 female and 290 male mice from MGP Select (breeders chow), and 363 female and 370 male mice from Mouse GP (HFD). The experiment was not replicated, rather the analyses incorporates data from a large time period encompassing many animals and litters.

DEXA was performed alongside the tests for auditory brainstem response and whole body, high-resolution digital X-ray. Mice were anaesthetised with ketamine hydrochloride. Nose to tail base length measurements were performed using a ruler with 1 mm graduations prior to DEXA measurement. Body composition parameters were measured using a PIXImus densitometer in combination with Lunar PIXImus2 2.1 software (GE Lunar, Madison, WI, USA).

Intraperitoneal glucose tolerance test: mice were single housed and fasted overnight (typically 16 h). A fasting (T0) blood sample from the tail tip was directly taken (Accu-chek Aviva, Roche, Indianapolis, IN, USA). Mice were then injected with 2 g kg^−1^ glucose intraperitoneally. Further blood samples were taken at 15 (T15), 30 (T30), 60 (T60) and 120 (T120) min post glucose injection. Area under the curve was calculated using the trapezoid method, where glucose at T0 was used as the baseline value for the mouse. Glucose clearance rate in mm min^−1^ was calculated by the formula (glucose at T120–glucose at T30)/90.

Clinical chemistry: blood was collected from animals in the random-fed state between 0830 and 1030. Mice were anesthetised using 100 mg kg^−1^ Ketamine and 10 mg kg^−1^ Xylazine, and blood was collected into heparinised pediatric tubes (Kabe Labortechnik GmbH, Numbrecht, Germany) using the retro-orbital route, followed by heart removal. Heparinised whole-blood samples were centrifuged at 5000 r.c.f. for 10 min at 4 °C, and the separated plasma analysed using an Olympus AU400 (High Wycombe, UK).

Further information about housing and husbandry, experimental procedures and pipeline quality control performed in the screen can be found in the Supplementary Material.

### Statistical and bioinformatics analysis

The individual mouse was considered the experimental unit within the studies. An iterative top down mixed modelling strategy was performed as described in Karp *et al.*^22^ using PhenStat version 2.3.2,^23^ an R package freely available from Bioconductor.^24^ The package's mixed model framework was used inputting diet as the genotype variable. The ACM (Equation 1) was obtained by setting the argument equation to ‘withoutWeight' and the WAM (Equation 2) by setting the argument to ‘withWeight'.


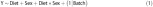



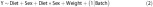


The reference group was the breeders chow (MGP Select) female mice, and hence model estimates for ‘Sex' are for the impact of being male and for ‘Diet' the impact of the HFD. The covariance structure for the residual is assessed, and if significant (*P*<0.05), then the model uses a heterogeneous model to account for the variance depending on diet. PhenStat uses a model optimisation strategy, and therefore, if weight as a covariate is not found to be a significant source of variation, it is dropped from the modelling process, and in effect, the WAM is equivalent to the ACM. The appropriateness of the models was assessed by visual inspection of graphic tools provided with PhenStat. In Equation 2, where weight is included as a covariate, the model adjusts for this variable by estimating the linear relationship between the covariate and the variable of interest, and as such makes two assumptions. First, that there is a linear relationship between the confounding variable and the variable of interest, and second, the assumption that a common linear relationship exists across treatment groups (homogeneity of regression slope). These assumptions can be assessed by plotting the confounding variable (body weight) against the outcome (phenotypic measure) and comparing the gradients on regression lines fitted for each experimental condition (pipeline). This was inspected for each variable, and this assumption was found to not be valid for five variables (log(alanine transaminase), log(aspartate aminotransferase), alkaline phosphatase, low-density lipoprotein cholesterol and free cholesterol). In these situations, the WAM was not appropriate. For these variables, we explored the data and the relationship with weight using a model that allowed for an interaction, and as the relationship was complex and the effect of diet could not be separated from weight, no weight-adjusted analysis was completed.

The appropriateness of the model output was also assessed by inspection of normal quantile–quantile plots of the residuals, a box plot that assessed the distribution of the residuals with batch by diet, and a plot that compares the residuals against predicted for each diet. From this analysis, four variables (insulin, creatine kinase, aspartate aminotransferase and alanine transaminase) were found to have issues with the ‘normality of residual' assumption when fitted with Equation 1 and Equation 2. A log10 transformation addressed this issue.

Percentage change was calculated to allow comparison of the diet effect for each sex across variables by taking the estimated coefficient from the regression analysis and dividing it by the average signal seen for that variable. Multiple testing was managed by controlling the family-wise error rate to 5% using the Holm method.^25^

### Data access and code availability

As a reference data set set for these animal models, we have made the data freely available via Zenodo.^21^ As a reproducible analysis, we also provide the scripts and output data that generated the results presented within the manuscript.^21^

## Results

### The effect of HFD, independent of sex and body weight

First, we investigated the impact of the HFD, assessing for absolute changes in phenotypes independent of sex and body weight differences by utilising the ACM (Table 2, Supplementary Table 1). Body weight on average increased by 24% at the three time points studied. For the other 38 variables examined, all were statistical significantly different. With large group size of mice contributing to the overall power to detect change of small effect size, the effect size for a given parameter could be small. The average largest effect seen across the two sexes for each variable was 29% with a range of 1.5–135% (s.d.: 28.1%). If we arbitrarily select a 5% threshold for biologically significance, then 87% (33/38) of the variables were significantly affected by diet. As expected for a HFD, glucose tolerance decreased, the body composition was primarily affected by a substantial increase in fat mass, and in plasma chemistry, most variables related to lipid and protein metabolism were increased, however, plasma triglycerides, creatinine and urea were decreased.

### HFD affects phenotypes in a sexually dimorphic manner

**Figure 1:**
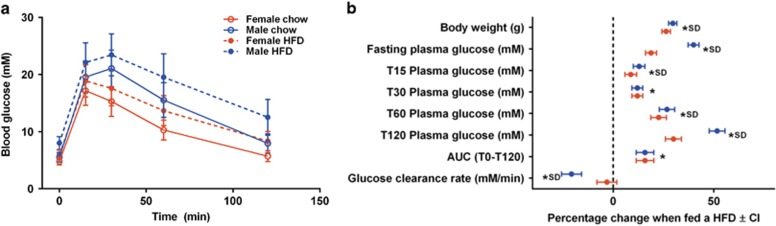
The estimated effect of a HFD on glucose tolerance for B6N mice. Glucose tolerance measures on B6N mice collected from an ipGTT screen implemented at the high-throughput phenotyping project at the WTSI. HFD data: 296 female and 290 male mice; Breeder's chow: 363 female and 370 male mice. (**a**) The blood glucose levels as a function of time during the ipGTT screen. The error bars indicate s.d. (**b**) The estimated effect of the HFD for each sex (blue for male and red for female) and for each variable associated with ipGTT screen with 95% confidence intervals from fitting an ACM. The star indicates statistical significance for the variables in a sex-independent manner and SD indicates the phenotype was classed as sexually dimorphic. CI, confidence interval; SD, sexually dimorphic; WTSI, Wellcome Trust Sanger Institute.

**Figure 2:**
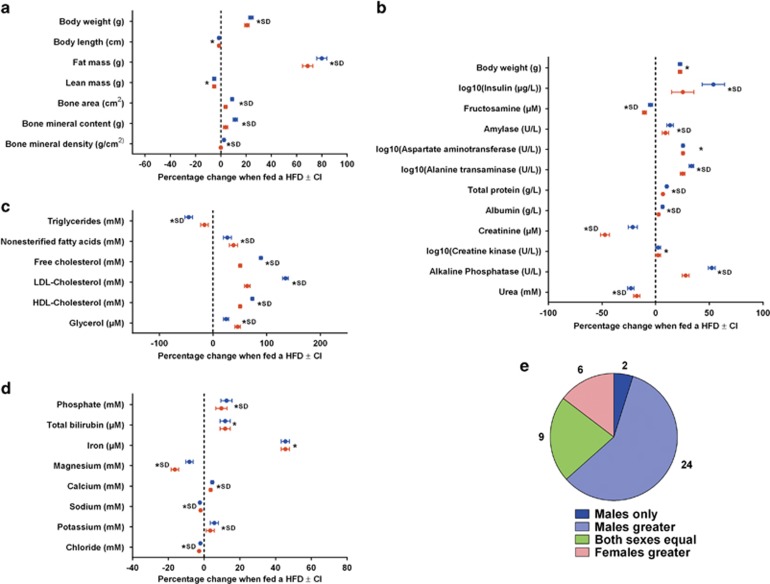
The estimated effect of the HFD from fitting an absolute change model and the role of sex in modifying the effect. Body composition and clinical chemistry measures on B6N mice collected from the high-throughput phenotyping project at the WTSI. Shown are outputs from fitted an absolute change model. HFD data: 296 female and 290 male mice; Breeder's chow: 363 female and 370 male mice. (**a–d**) The estimated effect of the HFD for each sex (blue for male and red for female) and for each variable. The error bars indicate the 95% confidence interval. The star indicates statistical significance and SD indicates the phenotype was classed as sexual dimorphic. (**a**) Body composition variables. (**b**) Proteins and enzyme variables. (**c**) Lipid variables. (**d**) Mineral variables. (**e**) The classification of how sex modified the effect of diet across all variables fitted with the absolute change model. SD, sexually dimorphic; WTSI, Wellcome Trust Sanger Institute.

Sex was found to be a statistically significant modifier of the effect of diet for the majority of traits (78%, 32/41) (Figures 1 and 2; Supplementary Table 1). By looking at the model estimates, the change observed could be classified according to outcome.^23^ For 22% (9/41) of variables, the effect was equal across the two sexes. For those classified as having a sexually dimorphic phenotype, the effect was dominated by male-specific/more pronounced male classifications (63.4%, 26/41) with the remainder being classified as female-specific/more pronounced female (14.6%, 6/41) (Figure 2). On average, for those variables classed as sexually dimorphic, there was a 13.2% difference in the effect size between the sexes (range: 0.42%–71.2%).

Glucose control, examined by ipGTT (Figure 1a), was affected in a sexually dimorphic manner. Male mice on HFD showed worsened blood glucose levels at almost all time points during the ipGTT compared to the female HFD-fed mice. Also the glucose clearance rate was only altered in the HFD males, while the HFD female had a glucose clearance rate that was comparable to the chow-fed females (Figure 1b). In terms of body composition, the major effect of HFD was elevated fat mass (Figure 2a), although sexually dimorphic effects were also noted for this and for bone parameters, with males being more affected than females. Regarding clinical chemistry, insulin levels were higher in male HFD mice compared to female HFD mice (Figure 2b), as were liver enzymes related to steatohepatitis, aspartate aminotransferase and alanine transaminase. In addition, HFD males also had worsened plasma lipid profiles, with higher cholesterol levels compared to the HFD females (Figure 2c).

### Inclusion of body weight as a covariate impacts the outcomes of metabolic phenotype analysis

**Figure 3:**
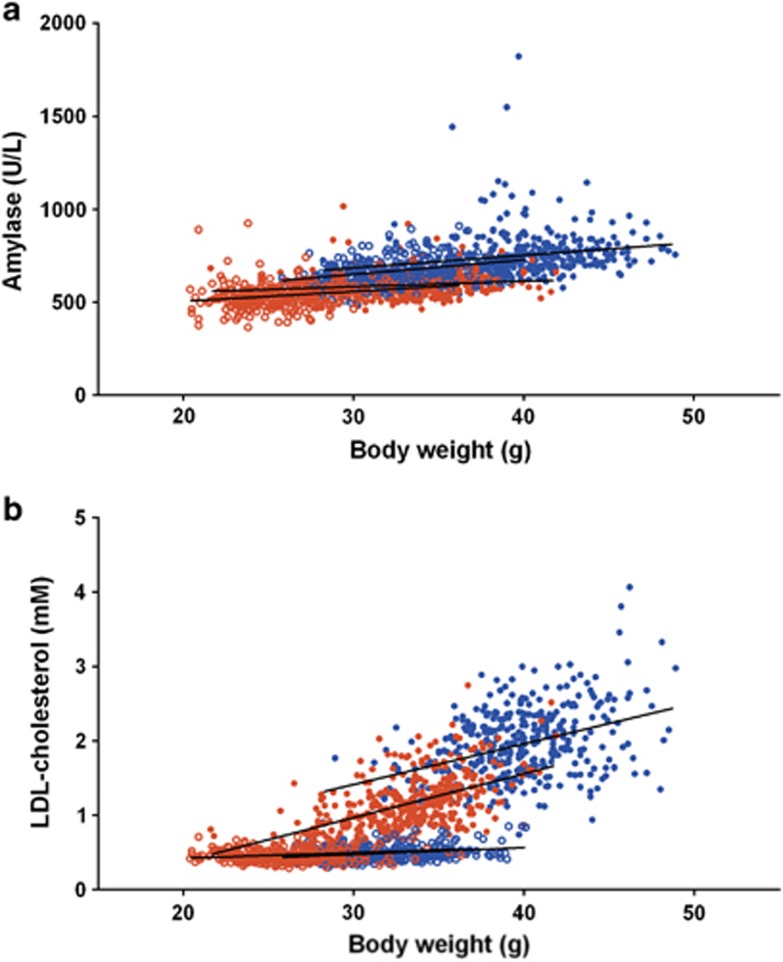
Example relationship between body weight and phenotypic trait of interest. The relationship between body weight and selected phenotypes observed for B6N mice from the high-throughput phenotyping project at the WTSI. HFD data: 296 female and 290 male mice; Breeder's chow: 363 female and 370 male mice. Shown in red are female mice and blue are male mice. Closed circles represent HFD-fed mice and open circles chow-fed animals. The fitted line is a regression line. (**a**) Amylase; (**b**) LDL cholesterol. LDL, low-density lipoprotein. WTSI, Wellcome Trust Sanger Institute.

We then assessed the relationship between the variables and body weight for the two diets. Visually, inspection of the relationship between body weight and diet, found five variables for which the relationship with weight depended on the diet (log(aspartate aminotransferase), alkaline phosphatase, free cholesterol, log(alanine transaminase) and low-density lipoprotein cholesterol). These variables positively correlated with body weight in HFD-fed mice, but not for the breeders chow-fed mice, despite overlap in the body weight ranges for these diets (Figure 3). This demonstrates selective, weight-independent effects of HFD on important and commonly measured physiological parameters. In addition, a positively correlated relationship was seen between body weight and both lean mass and fat mass, and the relationship was independent of diet (Supplementary Figure 1). Therefore, for the five variables, the differing relationship with weight is not due to an alternative relationship with lean or fat mass (Supplementary Figure 2). Therefore, as the effect of body size could not be separated from the effect of diet, these five variables were not carried forward for further analysis.

For the remaining variables, we assessed the effect of diet on the phenotype after adjusting for the confounding relationship with weight using WAM. This determined the body-weight-adjusted relative change in phenotype. With the model optimisation strategy implemented, weight as a covariate would only be included if it was a significant source of variation (likelihood ratio test *P*<0.05) and was included for 79% (26/33) of the variables.

**Figure 4:**
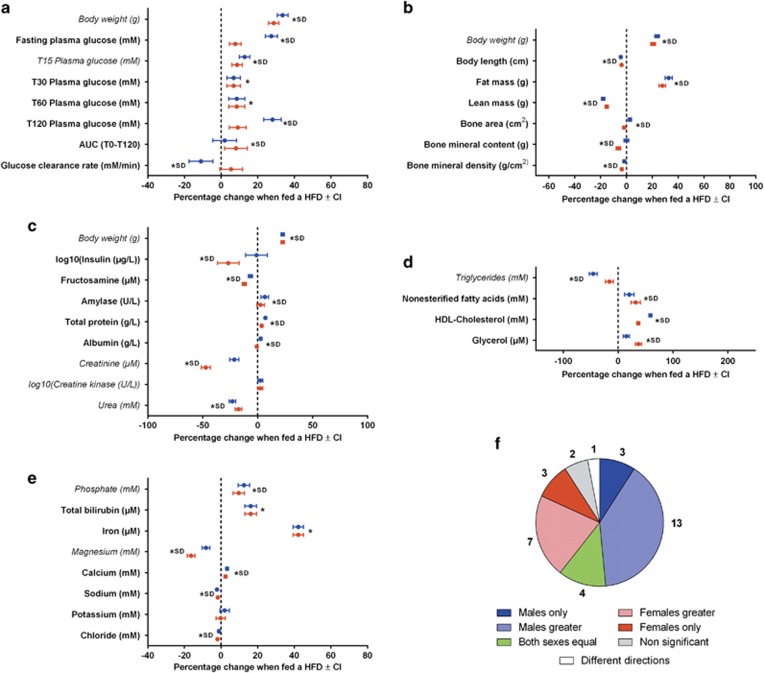
The estimated effect of the HFD from fitting a weight-adjusted model and the role of sex in modifying the effect. Outputs from fitting the WAM to phenotypic data collected for B6N mice from the high-throughput phenotyping project at the WTSI (excluding the weight variables fitted with ACM model). HFD data: *n*=296 female and 290 male mice; Breeder's chow: *n*=363 female and 370 male mice. (**a–d**) The estimated effect of the HFD for each sex (blue for male and red for female) for each variable. The error bars indicate the 95% confidence interval. The star indicates statistical significance and SD indicates the phenotype was classed as sexually dimorphic. Variables in italics did not show a confounding relationship with body weight. The ACM effect of HFD on body weight is shown for comparison—(**a**) Variables associated with the ipGTT screen. (**b**) Body composition variables. (**c**) Proteins and enzyme variables. (**d**) Lipid variables. (**e**) Mineral variables. (**f**) The classification of how sex modified the effect of diet across all variables fitted with the weight-adjusted model. AUC, area under the curve; HDL, high-density lipoprotein; SD, sexually dimorphic; WTSI, Wellcome Trust Sanger Institute.

Of the 33 variables assessed with the WAM model, the effect of HFD was significant in 93.9% (31/33), and of those that were significant, the average largest effect seen across the two sexes for these variables was 18.2% (range: 1.9–58.9%, s.d.: 15.7%). Including an additional arbitrary biological filter of 5% leads to 72.7% (24/33) of variables being classed as significantly different between the breeder's chow diet and HFD. In addition, of those classed as statistically significant, sex was found to be a modifier of the effect of diet for the majority of traits (81.8%, 27/33) (Figure 4). For just 12% (4/33), the effect was classed as ‘both sexes equally' as there was no evidence of an interaction between sex and diet, while for those identified as having a sexually dimorphic phenotype, the effect was dominated by male-specific/male stronger classifications (48.4%, 16/33) compared to female-specific/female stronger (30.3%, 10/33). One variable, DEXA bone area, was found to be sexually dimorphic, but with the direction of effect in opposite directions for each sex (Figure 4). On average, for those classed as sexually dimorphic, there was a 9.5% difference in the effect between the sexes (range: 0.42–29.4%).

Our analysis demonstrates that after accounting for body weight differences, the majority of variables were still significantly different between the breeder's chow diet and HFD. However, the average maximum effect size across the sexes was reduced (ACM: 29%, WAM: 18.2%). In addition, for sexually dimorphic variables, the inclusion of body weight as a covariate reduced the size of the difference seen between the sexes (ACM: 13.2%, WAM: 9.5).

## Discussion

We have used data generated from our high-throughput phenotyping pipeline to develop a core resource for research centred on the B6N mouse. In total, we use data on 41 metabolic-related variables from 1319 mice to produce a highly sensitive assessment of the impact of diet and sex on these variables. This extensive reference data set finds that the HFD has a wide-ranging impact on the metabolic variables, both when assessed for an absolute phenotypic difference and in the relative phenotypic effect where body weight is accounted for. While a number of papers have previously considered the metabolic profile of B6N mice,^1, 26, 27^ to date, little attention has considered the role of sex in the phenotype, particularly in conjunction with HFD.

Our analysis finds that sex is a significant modifier of the impact of HFD (78% of variables with the ACM) and is dominated by the males being affected to a greater degree than the females. The finding that sex is a significant modifier, supports the call^4, 5, 10, 28^ to always consider sex when designing and analysing *in vivo* studies. Indeed, sexual dimorphism in metabolic disorders such as obesity^29^ and atherosclerosis^30^ are well described and biologically important element of understanding their aetiologies. However, most *in vivo* studies focus on male mice.^4^ Historically, females were thought to have greater variability due to the oestrogen cycle, this however has been largely disproven.^31, 32^ In addition, previous studies have noted that the development of obesity and hyperglycaemia is strain-dependent and dependent on sex.^33, 34^ The extent of sexual dimorphism seen in this data set, combined with previous examples of phenotypic outcomes depending on sex, demonstrate the importance of assessing the role of sex when conducting research to understand disease progression, severity and treatment.

In general, many of the global findings on weight and glucose control for the effect of HFD on C57BL/6N observed were similar to those previously published.^35, 36, 37^ Our comprehensive analysis also identifies additional variables that are affected by HFD in the C57BL/6N background. The challenge with using mouse models for complex diseases, such as obesity and the metabolic syndrome, is that certain phenotypic outcomes may not fully recapitulate all aspects of the human disease. For example, we find that plasma iron levels are reduced in C57BL/6N HFD-fed mice, which matches the general observation that obesity corresponds with iron deficiency in humans.^38^ However, markers of elevated iron stores such as hyperferritinemia occur in a subset of metabolic syndrome patients and has been termed ‘dysmetabolic iron overload syndrome'.^39^ Similarly, bilirubin levels are inversely correlated with prevalence of type 2 diabetes in humans,^40^ and mouse models of insulin resistance are protected by haem oxygenase-induced production of bilirubin.^41^ However, increased haem oxygenase-1 expression is associated with insulin resistance and loss of haem oxygenase-1 also appears to be protective against metabolic disorders in mouse.^42^ In our analysis, bilirubin levels are elevated in HFD-fed mice, suggesting that the haem oxygenase-bilirubin pathway may be enhanced, thereby altering the baseline that the effect of interventions must be measured from. Our data therefore suggest that the design and interpretation of studies using HFD-fed mice to model human diseases should be careful evaluated.

In addition to the high prevalence of sexual dimorphism, our analysis found that with diet impacted most variables both when assessing the phenotypic change in absolute and relative terms. The use of a body weight correction by regression has been proposed for certain metabolic assays, for example, indirect calorimetry measurements of energy expenditure^15^ or body composition measures.^43^ A dual analysis, assessing both absolute and relative phenotypic changes, gives a richer understanding of the treatment. Bone mineral density (BMD), is one variable where published analysis has frequently considered the animal size as a co-variable. In humans, obesity is thought to increase BMD by mechanical loading, but also increases fracture risk.^44^ However, other studies point to an inverse relationship with BMD.^45, 46^ These conflicting observations highlight the complexity of the relationship, an observation that is also seen in the mouse literature. For example Li *et al.*,^47^ studying multiple inbred mouse strains, concluded that the relationship between BMD and body composition, particularly fat mass, is dependent on the diet used. They also reported that while sex had no net effect on BMD, once body weight is taken into account, females have a higher BMD than males. In comparison, using the ACM on our B6N mouse data, we find that HFD affects bone density in a sexually dimorphic manner, so that males have a greater response than females. In comparison, using the WAM to take body weight into account, we find the relative effect of HFD is an unchanged BMD in males but lowered BMD in females. As noted by Li *et al.* and others,^47, 48, 49^ the relationships between body composition, BMD, genetics, diet and environment are complex, and the experimental design, the model system and the specific variables and co-variables measured will play a significant part in the outcome from dietary interventions.

In summary, we have produced a reference data set for normal and HFD-fed C57BL6/N mice from a standardised phenotyping pipeline. We demonstrated that sexual dimorphism exists in the response to the diet in a large range of common metabolic variables, and suggest that analysis examining the effect of sex and body weight on phenotypes should be encouraged.

## Figures and Tables

**Table 1:** Diet composition of the breeders chow and the high-fat diet

*Component*	*Breeders chow (% g g^−1^) Mouse Breeder Diet 5021*	*High-fat diet (% g g^−1^) Western RD, 829100*
Crude protein	21.5	17.5
Crude fat	9	21.4
Cholesterol	2.76 × 10^−5^	~0.2
Crude fibre	3.5	3.5
Ash	5.5	4.1
Starch	34.3	5
Sugar	1.1	33.9

**Table 2:** Summary of the impact of the HFD on B6N mice as an absolute change in phenotype after controlling the family-wise error rate to 5% using data collected from the WTSI high-throughput phenotyping pipeline

*Screen*	*Increased variables*	*Decreased variables*
ipGTT	Fasting plasma glucose	Glucose clearance rate
	T15 plasma glucose	
	T30 plasma glucose	
	T60 plasma glucose	
	T120 plasma glucose	
	ipGTT area under curve	
DEXA	Fat mass	Lean mass
	Bone area	Body length
	Bone mineral content	
	Bone mineral density	
Plasma chemistry	Log10(insulin)	Fructosamine
	Amylase	Creatinine
	Log10(aspartate aminotransferase)	Urea
	Log10(alanine transaminase)	Triglycerides
	Total protein	Magnesium
	Albumin	Sodium
	Alkaline phosphatase	Chloride
	Non-esterified fatty acids	
	Free cholesterol	
	Low-density lipoprotein cholesterol	
	High-density lipoprotein cholesterol	
	Glycerol	
	Phosphate	
	Total bilirubin	
	Iron	
	Calcium	
	Potassium	
	Log10(creatine kinase)	

Abbreviations: DEXA, dual-energy X-ray absorptiometry; HFD, high-fat diet; ipGTT, intraperitoneal glucose tolerance test; WTSI, Wellcome Trust Sanger Institute.

## References

[bib1] Podrini C, Cambridge EL, Lelliott CJ, Carragher DM, Estabel J, Gerdin AK et al. High-fat feeding rapidly induces obesity and lipid derangements in C57BL/6N mice. Mamm Genome 2013; 24: 240–251. 23712496 10.1007/s00335-013-9456-0 PMC3685703

[bib2] Skarnes WC, Rosen B, West AP, Koutsourakis M, Bushell W, Iyer V et al. A conditional knockout resource for the genome-wide study of mouse gene function. Nature 2011; 474: 337–342. 21677750 10.1038/nature10163 PMC3572410

[bib3] Montgomery MK, Hallahan NL, Brown SH, Liu M, Mitchell TW, Cooney GJ et al. Mouse strain-dependent variation in obesity and glucose homeostasis in response to high-fat feeding. Diabetologia 2013; 56: 1129–1139. 23423668 10.1007/s00125-013-2846-8

[bib4] Beery AK, Zucker I. Sex bias in neuroscience and biomedical research. Neurosci Biobehav Rev 2011; 35: 565–572. 20620164 10.1016/j.neubiorev.2010.07.002 PMC3008499

[bib5] Yoon DY, Mansukhani NA, Stubbs VC, Helenowski IB, Woodruff TK, Kibbe MR. Sex bias exists in basic science and translational surgical research. Surgery 2014; 156: 508–516. 25175501 10.1016/j.surg.2014.07.001

[bib6] Geller SE, Koch A, Pellettieri B, Carnes M. Inclusion, analysis, and reporting of sex and race/ethnicity in clinical trials: have we made progress? J Womens Health 2011; 20: 315–320. 10.1089/jwh.2010.2469 PMC3058895 21351877

[bib7] Shen MC, Zhao X, Siegal GP, Desmond R, Hardy RW. Dietary stearic acid leads to a reduction of visceral adipose tissue in athymic nude mice. PLoS ONE 2014; 9: e104083. 25222131 10.1371/journal.pone.0104083 PMC4164353

[bib8] Zhang ZQ, He LP, Liu YH, Liu J, Su YX, Chen YM. Association between dietary intake of flavonoid and bone mineral density in middle aged and elderly Chinese women and men. Osteoporos Int 2014; 25: 2417–2425. 25062726 10.1007/s00198-014-2763-9

[bib9] Liu DM, Lu N, Zhao L, Zhang MJ, Tao B, Xuan Y et al. Serum Sema3A is in a weak positive association with bone formation marker osteocalcin but not related to bone mineral densities in postmenopausal women. J Clin Endocr Metab 2014; 99: E2504–E2509. 25050899 10.1210/jc.2014-1443

[bib10] Flanagan KL. Sexual dimorphism in biomedical research: a call to analyse by sex. Trans R Soc Trop Med Hyg 2014; 108: 385–387. 24934286 10.1093/trstmh/tru079

[bib11] Pettersson US, Walden TB, Carlsson PO, Jansson L, Phillipson M. Female mice are protected against high-fat diet induced metabolic syndrome and increase the regulatory T cell population in adipose tissue. PLoS ONE 2012; 7: e46057. 23049932 10.1371/journal.pone.0046057 PMC3458106

[bib12] Nishikawa S, Yasoshima A, Doi K, Nakayama H, Uetsuka K. Involvement of sex, strain and age factors in high fat diet-induced obesity in C57BL/6J and BALB/cA mice. Exp Anim 2007; 56: 263–272. 17660680 10.1538/expanim.56.263

[bib13] El Akoum S, Lamontagne V, Cloutier I, Tanguay JF. Nature of fatty acids in high fat diets differentially delineates obesity-linked metabolic syndrome components in male and female C57BL/6J mice. Diabetol Metab Syndr 2011; 3: 34. 22166251 10.1186/1758-5996-3-34 PMC3277487

[bib14] Grove KL, Fried SK, Greenberg AS, Xiao XQ, Clegg DJ. A microarray analysis of sexual dimorphism of adipose tissues in high-fat-diet-induced obese mice. Int J Obes 2010; 34: 989–1000. 10.1038/ijo.2010.12 PMC3667412 20157318

[bib15] Tschop MH, Speakman JR, Arch JR, Auwerx J, Bruning JC, Chan L et al. A guide to analysis of mouse energy metabolism. Nat Methods 2012; 9: 57–63. 10.1038/nmeth.1806 PMC3654855 22205519

[bib16] Reed DR, Lawler MP, Tordoff MG. Reduced body weight is a common effect of gene knockout in mice. BMC Genet 2008; 9: 4. 18182103 10.1186/1471-2156-9-4 PMC2263071

[bib17] Karp NA, Segonds-Pichon A, Gerdin AKB, Ramirez-Solis R, White JK. The fallacy of ratio correction to address confounding factors. Lab Anim 2012; 46: 245–252. 22829707 10.1258/la.2012.012003 PMC4152922

[bib18] Oellrich A, Meehan TF, Parkinson H, Sarntivijai S, White JK, Karp NA. Reporting phenotypes in mouse models when considering body size as a potential confounder. J Biomed Semantics 2016; 7: 2. 26865945 10.1186/s13326-016-0050-8 PMC4748495

[bib19] Brown SD, Moore MW. Towards an encyclopaedia of mammalian gene function: the International Mouse Phenotyping Consortium. Dis Model Mech 2012; 5: 289–292. 22566555 10.1242/dmm.009878 PMC3339821

[bib20] White JK, Gerdin AK, Karp NA, Ryder E, Buljan M, Bussell JN et al. Genome-wide generation and systematic phenotyping of knockout mice reveals new roles for many genes. Cell 2013; 154: 452–464. 23870131 10.1016/j.cell.2013.06.022 PMC3717207

[bib21] Ingvorsen C, Karp NA, Lelliott CJ. Supporting data: the role of sex and body weight in metabolic effects of high fat diet on C57BL/6N mice, 2017. Available at: http://doi.org/10.5281/zenodo.61231. 10.1038/nutd.2017.6 PMC5436097 28394359

[bib22] Karp NA, Melvin D, Mott RF, Sanger Mouse Genetics Project. Robust and sensitive analysis of mouse knockout phenotypes. PLoS One 2012; 7: e52410. 23300663 10.1371/journal.pone.0052410 PMC3530558

[bib23] Kurbatova N, Mason JC, Morgan H, Meehan TF, Karp NA. PhenStat: a tool kit for standardized analysis of high throughput phenotypic data. PLoS One 2015; 10: e0131274. 26147094 10.1371/journal.pone.0131274 PMC4493137

[bib24] Gentleman RC, Carey VJ, Bates DM, Bolstad B, Dettling M, Dudoit S et al. Bioconductor: open software development for computational biology and bioinformatics. Genome Biol 2004; 5: R80. 15461798 10.1186/gb-2004-5-10-r80 PMC545600

[bib25] Holm S. A simple sequentially rejective multiple test procedure. Scand J Stat 1979; 6: 65–70.

[bib26] Simon MM, Greenaway S, White JK, Fuchs H, Gailus-Durner V, Wells S et al. A comparative phenotypic and genomic analysis of C57BL/6J and C57BL/6N mouse strains. Genome Biol 2013; 14: R82. 23902802 10.1186/gb-2013-14-7-r82 PMC4053787

[bib27] Rendina-Ruedy E, Hembree KD, Sasaki A, Davis MR, Lightfoot SA, Clarke SL et al. A comparative study of the metabolic and skeletal response of C57BL/6J and C57BL/6N mice in a diet-induced model of type 2 diabetes. J Nutr Metab 2015; 2015: 758080. 26146567 10.1155/2015/758080 PMC4469802

[bib28] Woodruff TK. Sex, equity, and science. Proc Natl Acad Sci USA 2014; 111: 5063–5064. 24715722 10.1073/pnas.1404203111 PMC3986183

[bib29] Palmer BF, Clegg DJ. The sexual dimorphism of obesity. Mol Cell Endocrinol 2015; 402: 113–119. 25578600 10.1016/j.mce.2014.11.029 PMC4326001

[bib30] Wang X, Magkos F, Mittendorfer B. Sex differences in lipid and lipoprotein metabolism: it's not just about sex hormones. J Clin Endocrinol Metab 2011; 96: 885–893. 21474685 10.1210/jc.2010-2061 PMC3070248

[bib31] Meziane H, Ouagazzal AM, Aubert L, Wietrzych M, Krezel W. Estrous cycle effects on behavior of C57BL/6J and BALB/cByJ female mice: implications for phenotyping strategies. Genes Brain Behav 2007; 6: 192–200. 16827921 10.1111/j.1601-183X.2006.00249.x

[bib32] Mogil JS, Chanda ML. The case for the inclusion of female subjects in basic science studies of pain. Pain 2005; 117: 1–5. 16098670 10.1016/j.pain.2005.06.020

[bib33] Leiter EH, Chapman HD, Coleman DL. The influence of genetic background on the expression of mutations at the diabetes locus in the mouse. 5. Interaction between the Db gene and hepatic sex steroid sulfotransferases correlates with gender-dependent susceptibility to hyperglycemia. Endocrinology 1989; 124: 912–922. 2912706 10.1210/endo-124-2-912

[bib34] Goren HJ, Kulkarni RN, Kahn CR. Glucose homeostasis and tissue transcript content of insulin signaling intermediates in four inbred strains of mice: C57BL/6, C57BLKS/6, DBA/2, and 129X1. Endocrinology 2004; 145: 3307–3323. 15044376 10.1210/en.2003-1400

[bib35] West DB, Boozer CN, Moody DL, Atkinson RL. Dietary obesity in nine inbred mouse strains. Am J Physiol 1992; 262(6 Pt 2): R1025–R1032. 1621856 10.1152/ajpregu.1992.262.6.R1025

[bib36] Berglund ED, Li CY, Poffenberger G, Ayala JE, Fueger PT, Willis SE et al. Glucose metabolism *in vivo* in four commonly used inbred mouse strains. Diabetes 2008; 57: 1790–1799. 18398139 10.2337/db07-1615 PMC2453626

[bib37] Lecka-Czernik B, Stechschulte LA, Czernik PJ, Dowling AR. High bone mass in adult mice with diet-induced obesity results from a combination of initial increase in bone mass followed by attenuation in bone formation; implications for high bone mass and decreased bone quality in obesity. Mol Cell Endocrinol 2015; 410: 35–41. 25576855 10.1016/j.mce.2015.01.001

[bib38] Aigner E, Feldman A, Datz C. Obesity as an emerging risk factor for iron deficiency. Nutrients 2014; 6: 3587–3600. 25215659 10.3390/nu6093587 PMC4179177

[bib39] Datz C, Felder TK, Niederseer D, Aigner E. Iron homeostasis in the metabolic syndrome. Eur J Clin Invest 2013; 43: 215–224. 23289518 10.1111/eci.12032

[bib40] Ohnaka K, Kono S, Inoguchi T, Yin G, Morita M, Adachi M et al. Inverse associations of serum bilirubin with high sensitivity C-reactive protein, glycated hemoglobin, and prevalence of type 2 diabetes in middle-aged and elderly Japanese men and women. Diabetes Res Clin Pract 2010; 88: 103–110. 20083320 10.1016/j.diabres.2009.12.022

[bib41] Li M, Kim DH, Tsenovoy PL, Peterson SJ, Rezzani R, Rodella LF et al. Treatment of obese diabetic mice with a heme oxygenase inducer reduces visceral and subcutaneous adiposity, increases adiponectin levels, and improves insulin sensitivity and glucose tolerance. Diabetes 2008; 57: 1526–1535. 18375438 10.2337/db07-1764

[bib42] Jais A, Einwallner E, Sharif O, Gossens K, Lu TT, Soyal SM et al. Heme oxygenase-1 drives metaflammation and insulin resistance in mouse and man. Cell 2014; 158: 25–40. 24995976 10.1016/j.cell.2014.04.043 PMC5749244

[bib43] Goran MI, Allison DB, Poehlman ET. Issues relating to normalization of body fat content in men and women. Int J Obes Relat Metab Disord 1995; 19: 638–643. 8574274

[bib44] Reid IR. Fat and bone. Arch Biochem Biophys 2010; 503: 20–27. 20599663 10.1016/j.abb.2010.06.027

[bib45] Aguirre L, Napoli N, Waters D, Qualls C, Villareal DT, Armamento-Villareal R. Increasing adiposity is associated with higher adipokine levels and lower bone mineral density in obese older adults. J Clin Endocrinol Metab 2014; 99: 3290–3297. 24878039 10.1210/jc.2013-3200 PMC4154102

[bib46] Cawsey S, Padwal R, Sharma AM, Wang X, Li S, Siminoski K. Women with severe obesity and relatively low bone mineral density have increased fracture risk. Osteoporos Int 2015; 26: 103–111. 25182230 10.1007/s00198-014-2833-z

[bib47] Li R, Svenson KL, Donahue LRB, Peters LL, Churchill GA. Relationships of dietary fat, body composition, and bone mineral density in inbred mouse strain panels. Physiol Genomics 2008; 33: 26–32. 18230669 10.1152/physiolgenomics.00174.2007

[bib48] Lecka-Czernik B, Stechschulte LA, Czernik PJ, Dowling AR. High bone mass in adult mice with diet-induced obesity results from a combination of initial increase in bone mass followed by attenuation in bone formation; implications for high bone mass and decreased bone quality in obesity. Mol Cell Endocrinol 2015; 410: 35–41. 25576855 10.1016/j.mce.2015.01.001

[bib49] Doucette CR, Horowitz MC, Berry R, MacDougald OA, Anunciado-Koza R, Koza RA et al. A high fat diet increases bone marrow adipose tissue (MAT) but does not alter trabecular or cortical bone mass in C57BL/6J mice. J Cell Physiol 2015; 230: 2032–2037. 25663195 10.1002/jcp.24954 PMC4580244

