# Supplementary material for: The role of sex and body weight on the metabolic effects of high-fat diet in C57BL/6N mice
Source: Nutr Diabetes. 2017 Apr 10;7(4):e261–. doi: 10.1038/nutd.2017.6 (PMC5436097; doi:10.1038/nutd.2017.6)
Supplement: Supplementary Material [file nutd20176x1.docx]

**Supplementary Material**

**The role of sex and body weight in metabolic effects of high fat diet on C57BL/6N mice.**

Authors: Camilla Ingvorsen*^1,2^, Natasha A. Karp*^3^, Christopher J. Lelliott^1^

* These authors contributed equally to this work

Affiliations:

^1^ Mouse Pipelines, Wellcome Trust Sanger Institute, Hinxton, Cambridge, UK, CB10 1SA

^2^ University of Cambridge Metabolic Research Laboratories, Wellcome Trust-MRC Institute of Metabolic Science, Addenbrooke’s Hospital, Robinson Way, Cambridge, UK, CB2 0QQ

^3^ Mouse Informatics Group, Wellcome Trust Sanger Institute, Hinxton, Cambridge, UK, CB10 1SA

**Supplementary Methods**

**Animals, housing and husbandry:**

A core B6N colony was set up using mice from external providers (Taconic Biosciences), and the core colony nucleus was actively refreshed at set generational points (typically 10 generations) and cryopreserved to avoid genetic drift. Mice were phenotyped in weekly batches of 7 males and 7 females as part of the standardised pipelines from the Mouse Genetics Project (MGP) at Wellcome Trust Sanger Institute (WTSI). Formal randomisation was not used to select mice for phenotyping; rather, each week B6N mice were selected for phenotyping by identifying the age-correct wildtype mice from the breeding colonies supplying mutants for high throughput phenotyping. Two cages per sex were used, ideally from the largest breeding colony at that time point. Mice phenotyped by the Mouse GP pipeline (January 2011 – February 2012) were given a HFD from 4 weeks of age (Western RD, 829100, 21.4% crude fat content, 42% kcal as fat,~0.2% cholesterol Special Diet Services, Witham, UK) ([1](#_ENREF_1)). Mice phenotyped by the MGP Select pipeline (February 2012-February 2013) were given a breeders chow (Mouse Breeder Diet 5021, 9% crude fat content, 21% kcal as fat, 0.276ppm cholesterol, Labdiet, London, UK) from weaning. Diet compositions are given in Table 1. All mice were given water and diet *ad libitum*, unless otherwise stated. Mice were maintained in a specific pathogen free unit on a 12hr light: 12hr dark cycle with lights off at 7:30pm and no twilight period. The ambient temperature was 21 ± 2^o^C and the humidity was 55 ± 10%. Mice were typically housed for phenotyping using a stocking density of 3-5 mice per cage (overall dimensions of caging: (L x W x H) 365 x 207 x 140mm, floor area 530cm^2^) in individually ventilated caging (Tecniplast Seal Safe1284L) receiving 60 air changes per hour. In addition to Aspen bedding substrate, standard environmental enrichment of two nestlets, a cardboard tunnel and three wooden chew blocks was provided.

The care and use of mice in the WTSI study was carried out in accordance with UK Home Office regulations, UK Animals (Scientific Procedures) Act of 1986 under two UK Home Office licences which approved this work (80/2076 and 80/2485) which were reviewed regularly by the WTSI Animal Welfare and Ethical Review Body. Animal welfare was assessed routinely for all mice involved. Adult mice were killed by terminal anaesthesia followed by exsanguination and either cervical dislocation or removal of the heart.

**Phenotyping Screen and Quality Control:**

The analysis uses data taken from a high-throughput phenotyping project, where a mouse is characterised by a series of standardised and validated set of tests underpinned by standard operating procedures ([www.mousephenotype.org/impress](http://www.mousephenotype.org/impress)), covering a variety of disease-related and biological systems, including the metabolic. For mouse management purposes, the cages have both genotype and allele information and hence the intraperitoneal glucose tolerance test (ipGTT) and Dual-energy X-ray absorptiometry (DEXA) screens are run unblinded. However, as a high throughput screen where genes are selected for study without hypothesis and mice are studied in multiple batches there is limited room for personal bias influences the results. The processing of blood samples for clinical chemistry assessment, were run blind using a barcode system. The data were obtained as detailed in ([1](#_ENREF_1)). Factors thought to affect the variables were standardised as far as possible. Where standardisation was not possible, steps were taken to reduce potential bias. For example, the MGP uses a “minimised operator” defined in the Mouse Experimental Design Ontology as “The process by which steps are taken to minimise the potential differences in the effector by training and monitoring of operator.” ([2](#_ENREF_2)). The data captured with the MEDO ontology can be accessed at <http://www.mousephenotype.org/about-impc/arrive-guidelines>.

Data from the following screens were included for analysis in this manuscript: overnight fast followed by ipGTT at 13 weeks of age; DEXA at 14 weeks of age; clinical chemistry at 16 weeks of age. The two phenotyping pipelines were identical with the exception that the Mouse GP has 4 additional screens (hair phenotyping, open field, hot plate and stress induced hypothermia tests). The analysis presented here assumes that the omission of these screens in the MGP Select pipeline does not impact the outcomes of the ipGTT, DEXA and clinical chemistry screens.

Dual-energy X-ray absorptiometry: DEXA was performed alongside the tests for auditory brainstem response and whole body, high resolution digital X-Ray. Mice were anaesthetised with ketamine hydrochloride (100 mg/kg, Ketaset®, Fort Dodge Animal Health)/xylazine hydrochloride (10 mg/kg, Rompun®, Bayer Animal Health). Nose to tail base length measurements were performed using a ruler with 1mm graduations prior to DEXA measurement. Body composition [fat mass (g), fat percentage estimate (%), lean mass (g), bone mineral density (g/cm^2^), and bone mineral content (g)] were measured using a PIXImus densitometer in combination with Lunar PIXImus2 2.1 software (GE Lunar, Madison, WI). Quality control was performed using a calibrated phantom before imaging.

Intraperitoneal Glucose tolerance test: Mice were single housed and fasted overnight (typically 16h). Approximately 0.5mm of the tail tip was removed with a scalpel blade and a fasting blood sample (T0) was directly taken (Accu-chek Aviva, Roche, Indianapolis, IN). Mice were then injected with 2g/kg glucose intraperitoneally and further blood samples were taken at 15 (T15), 30 (T30), 60 (T60) and 120 (T120) minutes post-glucose injection. Area under the curve (AUC) was calculated using the trapezoid method, where glucose at T0 was used as the baseline value for the mouse. Glucose clearance rate in mM/min was calculated by the formula (Glucose at T120-Glucose at T30)/90.

Clinical Chemistry (CC): Samples for clinical chemistry are routinely taken as part of the necropsy of the mice. Blood was collected from animals in the random-fed state between 08:30 and 10:30. Mice were anesthetized using 100 mg/kg Ketamine and 10 mg/kg Xylazine and blood was collected into heparinised paediatric tubes (Kabe Labortechnik GmbH, Numbrecht, Germany) using the retro-orbital route, followed by heart removal. Heparinised whole-blood samples were centrifuged at 5,000 rcf for 10 min at 4⁰C, and the plasma was collected and stored at 4⁰C until analysis, always within 1 hour of collection. Plasma variables were assessed at room temperature using an Olympus AU400. Glucose levels were not analysed from the clinical chemistry screen due to the rapid increase in plasma glucose under Ketamine/Xylazine based anaesthesia.

The pipelines were regularly monitored with Shewhart plots and run tests ([3](#_ENREF_3)) to visualise the data behaviour for each variable over time to identify potential issues with drift or systematic shifts. Although rarely occurring, when identified this was typically associated with changes in reagent manufacture processes or servicing of machines. Remedies were then taken to correct this. The analysis within this manuscript assumes no systematic shift occurring simultaneously with the diet change. Pre-set reasons are established for QC failures (e.g. insufficient plasma sample) and detailed within IMPRESS providing standardised options as agreed by area experts as to when data can be discarded. Data can only be QC failed from the dataset if clear technical reasons can be found for a measurement being an outlier. Reasons are provided and tracked within the database. Animals were excluded from the analysis in this manuscript if any of the chosen variables were QC failed, or if the animal was unable to complete the pipeline due to welfare issues. The final dataset contained 296 female and 290 male mice from MGP Select (breeders chow) and 363 female and 370 male mice from Mouse GP (high fat diet). The experiment was not replicated, rather the analyses incorporates data from a large time period encompassing many animals and litters.

**Supplementary Table 1: Summary of the analysis generated by Phenstat for both diets.**

This table contains two tabs for the analysis of the Breeder’s chow and HFD using the ACM model (tab 1) and the WAM model (tab 2). Each table gives the summary statistics for each variable, split by sex and diet (columns B-I) followed by the overall average for all mice included in the study (column J). This is followed by the statistical data generated by the Phenstat script. The final column refers to the phenotypic classification denoting whether there is sexual dimorphism present. Further information about the Phenstat output, including the latest released version of the R-script, can be found in the User’s Guide and Reference Manual at <https://bioconductor.org/packages/release/bioc/html/PhenStat.html>.

**Supplementary Figure 1: Relationship between body weight and lean or fat mass.**

The impact of diet for B6N mice on body composition. HFD data: n= 296 female and 290 male mice; Breeder’s chow: n= 363 female and 370 male mice. Triangles indicate HFD; circles, Breeder’s chow; red, female mice; and jade, male mice. The fitted line is a linear regression for each sex.


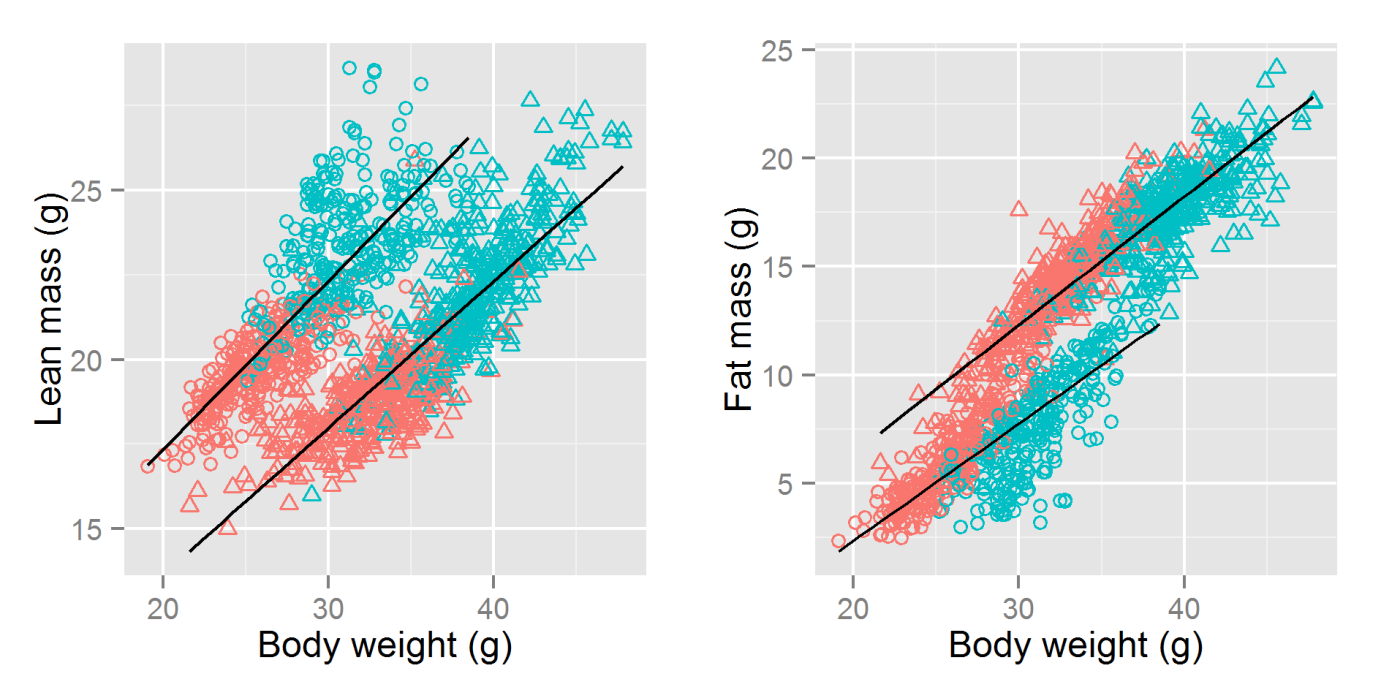


**Supplementary Figure 2: Relationship between body weight, lean and fat mass for various clinical chemistry variables.**

The impact of diet and body composition variable on selected clinical chemistry variables for B6N mice. HFD data: n= 296 female and 290 male mice; Breeder’s chow: n= 363 female and 370 male mice. Triangles indicate HFD; circles, Breeder’s chow; red, female mice; and jade, male mice. The fitted line is a linear regression.


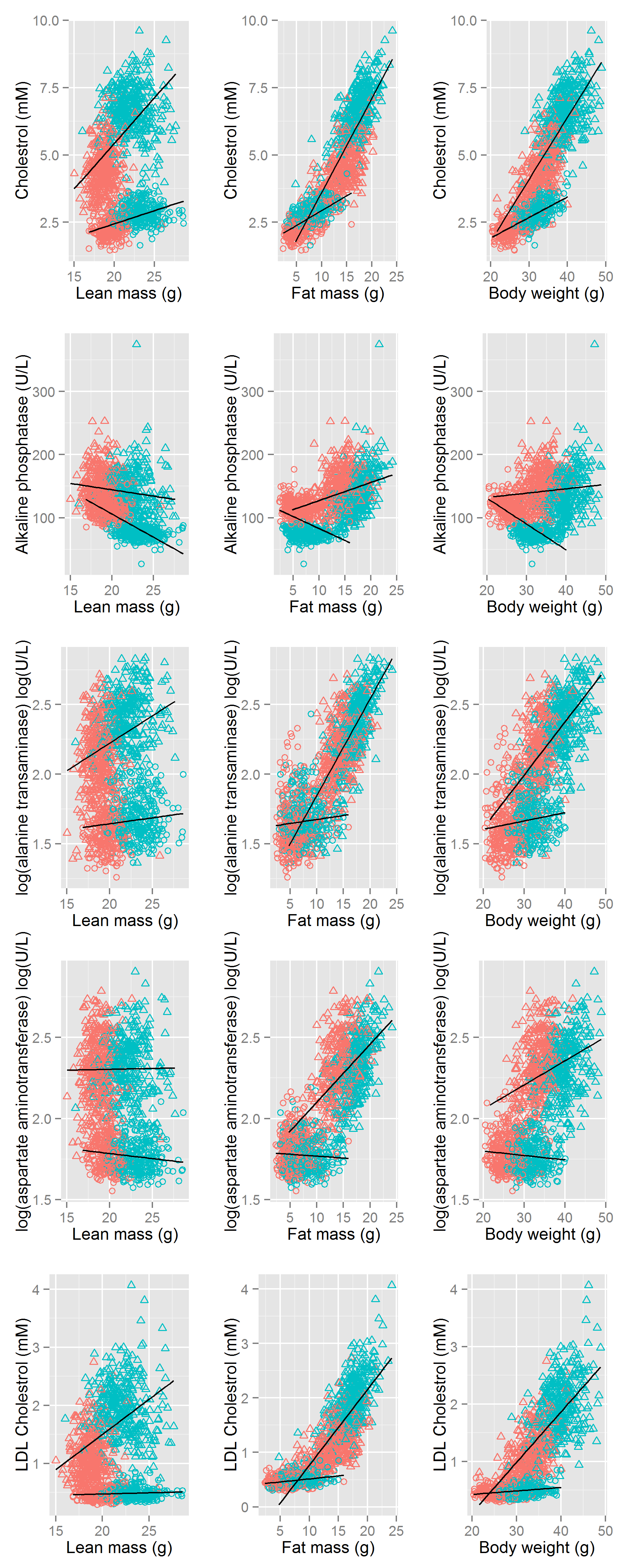


**References**

1. White JK, Gerdin AK, Karp NA, Ryder E, Buljan M, Bussell JN, et al. Genome-wide Generation and Systematic Phenotyping of Knockout Mice Reveals New Roles for Many Genes. Cell. 2013;154(2):452-64.

2. Karp NA, Meehan TF, Morgan H, Mason JC, Blake A, Kurbatova N, et al. Applying the ARRIVE Guidelines to an In Vivo Database. PLoS biology. 2015;13(5):e1002151.

3. Dixon W, Massey F. Sensitivity experiments. Introduction to Statistical Analysis 4th ed Boston: McGraw Hill. 1983:426-41.
